# Gut microbiome affects the response to anti-PD-1 immunotherapy in patients with hepatocellular carcinoma

**DOI:** 10.1186/s40425-019-0650-9

**Published:** 2019-07-23

**Authors:** Yi Zheng, Tingting Wang, Xiaoxuan Tu, Yun Huang, Hangyu Zhang, Di Tan, Weiqin Jiang, Shunfeng Cai, Peng Zhao, Ruixue Song, Peilu Li, Nan Qin, Weijia Fang

**Affiliations:** 1 Cancer Biotherapy Center, First Affiliated Hospital, School of Medicine, Zhejiang University, 79 Qingchun Road, Hangzhou, China; 2 Realbio Genomics Institute, 138 Xinjunhuan Road, Shanghai, China; 3 Shanghai Tenth People’s Hospital Affiliated to Tongji University, No. 301 Yanchangzhong Road, Shanghai, China; 4 Key Laboratory for Drug Evaluation and Clinical Research of Zhejiang Province, First Affiliated Hospital, School of Medicine, Zhejiang University, 79 Qingchun Road, Hangzhou, China; 5 Key Laboratory of Precision Diagnosis and Treatment for Hepatobiliary and Pancreatic Tumor of Zhejiang Province, First Affiliated Hospital, School of Medicine, Zhejiang University, 79 Qingchun Road, Hangzhou, China; 6 Zhejiang Provincial Key Laboratory of Pancreatic Disease, First Affiliated Hospital, School of Medicine, Zhejiang University, 79 Qingchun Road, Hangzhou, China

**Keywords:** Gut microbiome, Hepatocellular carcinoma, Anti-PD-1 immunotherapy

## Abstract

**Background:**

Checkpoint-blockade immunotherapy targeting programmed cell death protein 1 (PD-1) has recently shown promising efficacy in hepatocellular carcinoma (HCC). However, the factors affecting and predicting the response to anti-PD-1 immunotherapy in HCC are still unclear. Herein, we report the dynamic variation characteristics and specificities of the gut microbiome during anti-PD-1 immunotherapy in HCC using metagenomic sequencing.

**Results:**

Fecal samples from patients responding to immunotherapy showed higher taxa richness and more gene counts than those of non-responders. For dynamic analysis during anti-PD-1 immunotherapy, the dissimilarity of beta diversity became prominent across patients as early as Week 6. In non-responders, *Proteobacteria* increased from Week 3, and became predominant at Week 12. Twenty responder-enriched species, including *Akkermansia muciniphila* and *Ruminococcaceae* spp., were further identified. The related functional genes and metabolic pathway analysis, such as carbohydrate metabolism and methanogenesis, verified the potential bioactivities of responder-enriched species.

**Conclusions:**

Gut microbiome may have a critical impact on the responses of HCC patients treated with anti-PD-1 immunotherapy. The dynamic variation characteristics of the gut microbiome may provide early predictions of the outcomes of immunotherapy in HCC, which is critical for disease-monitoring and treatment decision-making.

**Electronic supplementary material:**

The online version of this article (10.1186/s40425-019-0650-9) contains supplementary material, which is available to authorized users.

## Introduction

With a high malignancy and a poor prognosis, hepatocellular carcinoma (HCC) is ranked as the fourth-leading cause of cancer-related fatalities worldwide, bringing a heavy burden to public health [1]. The oral multi-kinase inhibitor, sorafenib, is the standard systemic therapeutic option for advanced-stage HCC and has an objective response rate (ORR) of less than 5% [2]. Recently, checkpoint blockade immunotherapy targeting programmed cell death protein 1 (PD-1) has shown promising efficacy for treating HCC. In two multicenter, phase 2 studies [3, 4] investigating the efficacy of anti-PD-1 immunotherapy in sorafenib-refractory HCC, the ORR was nearly 20%, which quadrupled that of sorafenib. However, the factors affecting and predicting the response to anti-PD-1 immunotherapy in HCC are still unclear.

The role of the gut microbiome in modulating tumor responses to immunotherapy in melanoma [5–7], non-small cell lung cancer, renal cell carcinoma, and urothelial carcinoma [6, 8] has received increased attention across a series of studies in recent years. However, data concerning the impact of the gut microbiome on HCC immunotherapy have not been reported. Additionally, previous research to date has tended to focus more on the baseline status rather than a dynamic variation of the gut microbiome during immunotherapy. The aim of the present study, using fecal metagenomics as a snapshot, was to provide more specific understandings on how the gut microbiome influences the responses of HCC patients to anti-PD-1 immunotherapy.

## Results and discussion

Eight HCC patients with Barcelona Clinic Liver Cancer (BCLC) Stage C disease treated with anti-PD-1 antibodies after progression on sorafenib were enrolled in the present study. Anti-PD-1 antibodies were administered every 3 weeks. During the treatment, no antibiotics were applied. Patients were classified as responders (R, complete or partial response, or stable disease lasting for over 6 months; *n* = 3) and non-responders (NR, progressed disease or stable disease lasting less than 6 months; *n* = 5) based on radiological evaluation according to Response Evaluation Criteria in Solid Tumors (RECIST 1.1). Fecal samples were collected at the baseline (Day 0), Week 1 after treatment initiation, and every 3 weeks during therapy until disease progression, according to the informed consent and study protocol. Dynamic variation of gut bacterial characteristics was evaluated and analyzed by metagenomic sequencing.

**Fig. 1:**
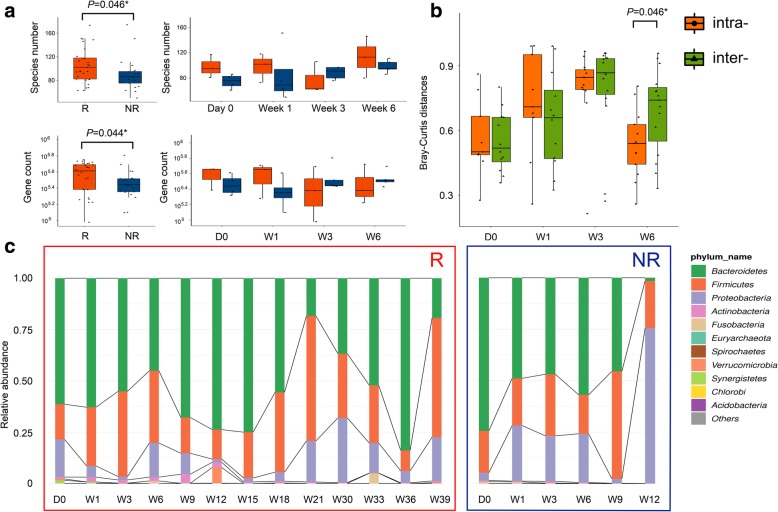
Difference in microbial diversity and composition between R and NR. **a** Alpha diversity measurements by species richness (up) and gene counts (down). Red: R; Blue: NR. **b** Beta diversity measurements, as indicated by intra- (orange) and inter-group (green) Bray-Curtis distances. **c** Microbial composition of R (left) and NR (right) at the phylum level. The ten most abundant phyla of each group are shown

Over the entire treatment, R showed higher taxa richness and more gene counts than those of NR (Fig. 1a). As for dynamic diversity analysis, the beta diversity evaluated by Bray-Curtis distances showed that the inter-group dissimilarity became significantly higher than the intra-group differentiation as early as Week 6 (Fig. 1b and Additional file 1: Figure S1). Dynamic microbial composition variation was also analyzed. Before treatment initiation, the Gram-positive *Firmicutes*, Gram-negative *Bacteroidetes* and Gram-negative *Proteobacteria* dominated the fecal microbiome of both R and NR, which was in accordance with the findings in healthy adults [9], suggesting that no severe gut microbiome dysbiosis was present in the study group at baseline. Specifically, *Bacteroidetes* were most abundant, followed by *Firmicutes* and *Proteobacteria*. As treatment proceeded, microbial composition at the phylum level in R remained relatively stable. However, in NR, *Proteobacteria* markedly increased as early as Week 3, and became predominant at Week 12 (Fig. 1c). Increase of *Proteobacteria* in NR was mainly attributed to the prevalence of *Escherichia coli*, while the most notable proteobacterial member in R was *Klebsiella pneumoniae*. Composition of *Bacteroides* and *Firmicutes* also exhibited individual patterns (Additional file 2: Figure S2 and Additional file 3: Figure S3). These findings suggested that the dynamic variation characteristics of gut-microbial diversity and composition at the early treatment period of anti-PD-1 immunotherapy in HCC may have distinct implications on drug efficacy and disease prognosis.

**Fig. 2:**
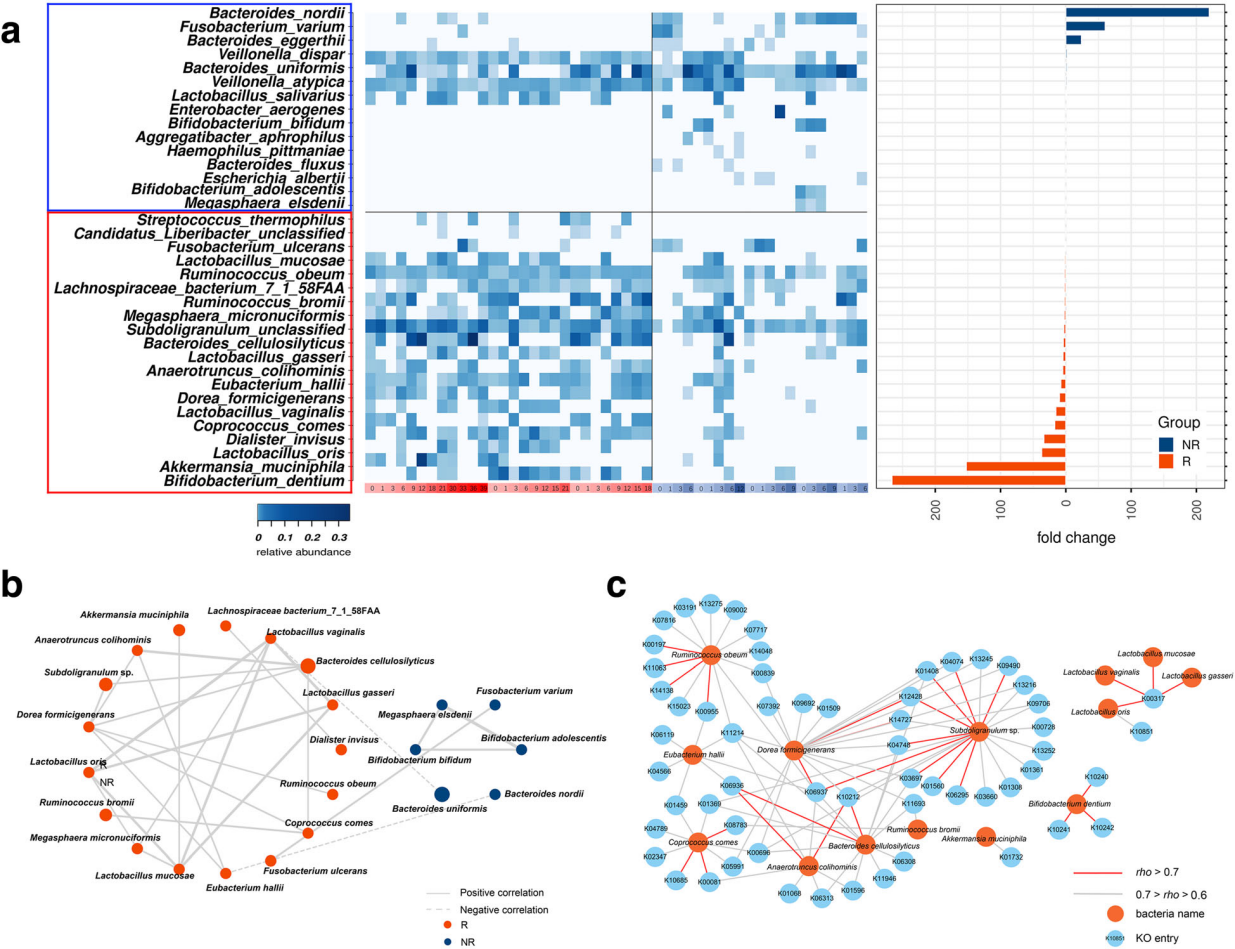
Meta-analysis of the bacteria significantly enriched in R and NR. **a** Heatmap showing the relative abundance of R-enriched and NR-enriched bacterial species, as identified by LEfSe. **b** Correlation network of the R-enriched and NR-enriched species (Spearman correlations with *rho* > 0.5, *P* < 0.01 were shown). The size of the nodes is proportional to the averaged relative abundance of these species in all samples. The thicknesses of the lines denote the strengths of the correlations. **c** Positive correlation network of the significant R-enriched species and KO categories

To further identify the species possibly affecting patient responses, linear discriminant analysis (LDA)-effect size (LEfSe) algorithm analysis was performed between all R and NR samples. Twenty R-enriched species and fifteen NR-enriched species were identified (Fig. 2a and Additional file 4: Figure S4). Among the R-enriched species, four *Lactobacillus* species (*L. oris*, *L. mucosae*, *L. gasseri*, and *L. vaginalis*), *Bifidobacterium dentium* and *Streptococcus thermophilus* were probiotic lactic acid bacteria, which were beneficial to host metabolism and immunity by inhibiting the growth of pathogenic microorganisms and accompanying spoilage agents. Oral administration of *Bifidobacterium* could improve tumor control efficacy of programmed cell death protein 1 ligand 1 (PD-L1)-specific antibody therapy [10]; *Coprococcus comes*, *Bacteroides cellulosilyticus*, and *Subdoligranulum* sp. also possessed probiotic potential, as they were reported to be related with dietary fiber digestion and short-chain fatty acid production. Notably, enrichment of one *Lachnospiraceae* and two *Ruminococcaceae* species (*Lachnospiraceae* bacterium 7_1_58FAA, *Ruminococcus obeum*, *Ruminococcus bromii*) and *Akkermansia muciniphila* in R was also observed. Commensal *A.muciniphila* and *Ruminococcaceae* benefited host health by preventing increases in intestinal permeability and systemic immunosuppression. In previous studies, a significantly higher relative abundance of *Ruminococcaceae* was identified in melanoma patients responding to anti-PD-1 immunotherapy [7], and oral supplementation with *A. muciniphila* could restore the efficacy of anti-PD-1 immunotherapy [8]. In the present study, the SparCC algorithm was applied to gain insights into the mutualistic networks between R-enriched and NR-enriched species (Fig. 2b). The number of significant positive-correlation pairs and the correlation strengths in R-enriched species were higher than those of NR-enriched species. Among the R-enriched bacteria, the four *Lactobacillus* species were most significantly correlated with each other, indicating their possible pivotal roles in the network. Our findings further indicated the biological significance of particular bacterial strains during anti-PD-1 immunotherapy in HCC and may provide support for the development of a gut-microbiome-modulation scheme in immunotherapy.

Next, functional gene families associated with R-enriched bacteria were investigated. A total of 189 Kyoto Encyclopedia of Genes and Genomes (KEGG) orthologies (KOs) were identified to be enriched in R (Kruskal-Wallis rank sum test, *P* < 0.05). Significant positive correlations were identified between 123 R-enriched KOs and 18 R-enriched species (Spearman’s correlation, *rho* > 0.5, Additional file 6: Table S1). Pathway identification of KOs verified the potential bioactivities of R-enriched species (Fig. 2c and Additional file 5: Figure S5). In detail, the cellobiose-transport system (ko02010) was significantly correlated with *B. dentium*; the pectin lyase (K01732), which may be involved in pectin metabolism, was correlated with *A. muciniphila*. Both cellulose and pectin have been highlighted for their prebiotic and anti-inflammatory potential as dietary fibers [11, 12]. Methanogenesis pathway (ko00680) was found to be correlated with *R. obeum* and the four *Lactobacillus* species. Methane generated in human gastrointestinal tract has been reported to ameliorate oxidative stress injury and suppress the host inflammatory response [13]. Other pathways with potential benefits included sulfate reduction (ko00920) and carbon fixation (ko00720) functions that were correlated with *R. obeum*, carotenoid biosynthesis (ko00906) correlated with *B. cellulosilyticus* and *A. colihominis*, and unsaturated fatty acid metabolism (ko00590) associated with *C. comes* (Additional file 7: Table S2). Such findings further illustrated the potential underlying mechanisms of gut microbiome influencing the anti-PD-1 immunotherapy efficacy in HCC patients.

It is well recognized that factors including age, genetics, and diet may influence microbiome composition [14]. However, the long-term stability of the human gut microbiota has been demonstrated in previous studies. A study demonstrated that an individual’s microbiota could be remarkably stable, with 60% of strains remaining over the course of five years; this finding highlighted that such stability and responsiveness to physiologic change confirmed the potential of the gut microbiota as a diagnostic tool and therapeutic target [15]. In the present study, patients were enrolled with strict criteria to minimize potential dietary or geographical influences during the whole course of treatment, as well as to ensure that the dynamic changes of gut microbiota in both R and NR were attributed to the therapeutic intervention instead of any daily factors.

Although our present microbiome-wide association study highlighted the association between changes in the gut microbiome and host-immune responses to drugs, any causal relationship of these correlative associations remains unknown. To further elucidate this relationship, several strategies have been developed. Firstly, ‘meta-omic’ analysis combining sequencing and multiple biochemical techniques can greatly advance the knowledge of the human microbiome and its specific role in governing disease states. For example, Gopalakrishnan et al. compared the tumor-associated immune infiltrates via multi-parameter immunohistochemistry (IHC) and found a statistically significant positive correlation between CD8+ T cell infiltrate in the tumor and the *Faecalibacterium* genus as well as the *Ruminococcaceae* family, suggesting a possible mechanism through which the gut microbiome may modulate anti-tumor immune responses [7]. More importantly, in vitro or in vivo experimental models are required in order to allow the systematic manipulation of variables and, thus, allow experimental testing and validation of results derived from meta-omics [16]. In our previous study, we had demonstrated that Behcet’s disease (BD), a kind of autoimmune disorder, was associated with considerable gut microbiome changes [17]. To determine whether the gut microbiome contributes to development of this disease, fecal microbiota transplantation (FMT) was performed in mice undergoing autoimmune uveitis. We demonstrated that mice colonized with whole-gut microbiomes from BD patients showed an exacerbation of disease activity and an excessive production of pro-inflammatory cytokines. These results may confirm the hypothesis that specific bacterial patterns contributed to the development of intraocular inflammatory disease. In our future studies, similar meta-omics strategies, as well as the use of mouse FMT models, will also be incorporated in order to deeply explore the interaction between the gut microbiome and host responses to immunotherapies among HCC patients.

Gopalakrishnan et al. also assessed the landscape of the oral and gut microbiomes in patients with metastatic melanoma receiving anti-PD-1 immunotherapy and demonstrated a high abundance of *Lactobacillales* in the oral microbiomes compared with that of fecal microbiomes in all subjects [7]. Galloway-Pena et al. further reported a high degree of intra-patient temporal instability of both stool and oral microbial diversity in patients with acute myeloid leukemia undergoing induction chemotherapy [18]. Both studies showed the possibility of using oral microbiome, rather than fecal microbiome, as an indicator for specific clinical outcomes. In our future longitudinal studies, both fecal and oral microbiome will be taken into consideration for a more comprehensive visualization of the host-microbe interactions.

In conclusion, by metagenomic sequencing of periodic fecal samples, we illustrated that the dynamic-variation characteristics of the gut microbiome might be used for early prediction of the six-month outcomes of anti-PD-1 immunotherapy in HCC at 3–6 weeks after treatment initiation, which is critical for disease monitoring and treatment decision-making. To our knowledge, this is the first study to focus on the association between the gut microbiome and the response to anti-PD-1 immunotherapy for HCC. Furthermore, while previous studies had mostly only provided cross-sectional comparisons, the present study showed the different trajectories of microbiome shifts between R and NR and revealed a stronger stability of the gut microbiome in R during the whole course of treatment. We believe that the potential of the gut microbiota as a therapeutic target is indicated in this study, and that the relevant bacterial species and metabolic pathways revealed here could be developed as a modulation strategy for better treatment options for HCC patients.

## Methods

### Patients and medications

A cohort of eight patients was included in this study. All patients were histologically confirmed HCC with BCLC Stage C disease and had experienced disease progression on first-line sorafenib treatment. Other eligibility criteria included radiological measurable disease, an adequate function of major organs, an Eastern Cooperative Oncology Group (ECOG) performance status of 0 or 1, and a Child-Pugh Class A liver function. Exclusion criteria included fibrolamellar HCC, sarcomatoid HCC, or mixed cholangiocarcinoma and HCC, liver transplantation, or a history of active autoimmune disease.

Patients received camrelizumab (SHR-1210, HengRui Medicine Co., Jiangsu, China), a humanized anti-PD-1 IgG4 monoclonal antibody, intravenously at a dose of 3 mg/kg every three weeks until disease progression or intolerance (Clinicaltrials.gov ID: NCT02989922). Written informed consent, including collection and analysis of microbiome samples, was obtained from each patient before initiation of the study. Patients were asked to keep their own dietary and other habits during drug treatment to avoid any interruption to their inherent gut microbiome. Additionally, in order to omit the geographical influence, we collected samples only from one hospital with local individuals who shared common dietary intake patterns. None of the patients had experienced diarrhea or other intestinal symptoms, or antibiotic/probiotic consumption during the study.

Radiological evaluation was assessed every six weeks according to the RECIST 1.1 criteria. Responders (R, *n* = 3) were defined by radiographic evidence as complete or partial response, or stable disease lasting for at least six months. Non-responders (NR, *n* = 5) were defined as those with progressed disease or stable disease lasting less than six months. This study complied with the Declaration of Helsinki and was approved by the Ethics Committee of the First Affiliated Hospital of Zhejiang University.

### Fecal sample collection

Fresh feces were collected on the last pre-treatment clinic visit (as baseline, Day 0), one week after treatment initiation, the day of each treatment and were stored at − 80 °C immediately prior to DNA extraction. According to the sample providing policy in the informed consent, the observation lasted for 39, 21, and 18 weeks for the three responders, and 6, 6, 9, 9, and 12 weeks for the five non-responders.

### DNA extraction and metagenomic sequencing

Dynamic variation of gut bacterial composition of R and NR during the anti-PD-1 immunotherapy was evaluated using fecal metagenomic sequencing. Briefly, bacterial genomic DNA was extracted using QIAamp DNA Stool Mini Kit (Qiagen, Hilden, Germany). After checking the integrity and concentration of DNA, individual libraries were constructed using MGIEasy DNA Library Prep Kit (BGI, Shenzhen, China), loaded into the BGISEQ-500 RS platform (BGI, Shenzhen, China), and were sequenced using 2 × 100 bp paired-end read protocol. The process of quality filtering, trimming, and demultiplexing was carried out as described previously [19]. A total of 49 datasets (28 from R and 21 from NR) were generated. Overall, 78.12% of the raw reads were regarded as high-quality reads, with an average length of 72 bp and an average Q35 score of 100% (Additional file 8: Table S3).

### Taxonomic and gene profiling

All high-quality reads were then aligned to *Homo sapiens* (human) genome assembly hg38 [20] using SOAPalign 2.21 with default parameters to remove human reads (https://anaconda.org/bioconda/soapaligner). The retained clean reads were then aligned to ~ 1 M clade-specific marker genes from approximately 17,000 reference genomes for estimation of relative phylotype abundance using MetaPhlAn (version 2.5.0) [21].

For gene annotation, the clean reads were aligned to the integrated gene catalog (IGC) [22] by using SOAPalign 2.21 with default parameters; only the reads with both ends mapped to the same gene were used in the following analysis. An average IGC mapping rate of 77.77% and an average unique mapping rate of 63.27% were achieved. The gene relative abundance profile was generated following the procedure described before [17]. Functional annotations were carried out by BLASTP search against the KEGG database (*e* value ≤1e − 5 and high-scoring segment pair scoring > 60) [23]. The KO abundance was estimated by accumulating the relative abundance of all genes belonging to this feature.

### Statistical analyses and correlation network

Non-parametric Wilcoxon rank-sum test was employed to analyze the statistical significance of diversity indices, taxa and KOs between R and NR. Bray-Curtis metrics were applied to calculate pairwise dissimilarities between samples and were used in beta diversity evaluation and principal coordinate analysis (PCoA) [24]. The LEfSe algorithm was applied to identify the phylotypes significantly different in relative abundance between all R and NR samples; phylotypes with an LDA score cut-off of 2.0 and *P* < 0.05 in built-in rank sum test were regarded as statistically significant [25].

SparCC algorithm was used to calculate correlations between R-enriched and NR-enriched species. Bootstrapping of 100 repetitions was used to compute the *P* value for each correlation, as described previously [17]. Only significant correlations with *P* < 0.05 and *rho* > 0.5 were presented in the network. Spearman correlation was applied to estimate the association strengths for determining the relationship between bacteria and KO categories. Only significant correlations with *P* < 0.01 and *rho* > 0.5 were presented in the network. The species-species network and the species-KO network were both visualized with Cytoscape3.0.2.

## Additional files

Additional file 1: **Figure S1.** Principal componeLnt analysis (PCoA) based on Bray-Curtis distances, followed by Adonis test at Day 0 (a), Week 1 (b), Week 3 (c), and Week 6 (d). (PNG 1455 kb)

Additional file 2: **Figure S2.** Microbial composition of the R (left) and NR (right) at the genus (a) and species (b) levels. The ten most abundant genera or species of each group are shown. (PNG 4920 kb)

Additional file 3: **Figure S3.** Sankey analysis of all R and NR during the treatment. (PNG 9946 kb)

Additional file 4: **Figure S4.** Differentially abundant genera (a) and species (b) between R and NR, identified by LEfSe. (PNG 2863 kb)

Additional file 5: **Figure S5.** Positive correlation network of significant R-enriched species and KOs. Correlations with *rho* > 0.7 are shown in red, while those with *rho* > 0.5 are shown in gray. (PNG 3679 kb)

Additional file 6: **Table S1.** List of significant positive correlations between species and KOs. (XLSX 27 kb)

Additional file 7: **Table S2.** Pathway assignment of the most significantly correlated KOs in R. (XLSX 14 kb)

Additional file 8: **Table S3.** Quality control of the sequencing data. (XLSX 19 kb)

## Data Availability

All sequencing datasets were uploaded to the NCBI database with SRA accession: PRJNA505228.

## Abbreviations

BCLC: Barcelona Clinic Liver Cancer
ECOG: Eastern Cooperative Oncology Group
HCC: hepatocellular carcinoma
IGC: integrated gene catalog
KEGG: Kyoto Encyclopedia of Genes and Genomes
KOs: Kyoto Encyclopedia of Genes and Genomes Orthologies
LDA: Linear discriminant analysis
LEfSe: Linear discriminant analysis - effect size
NR: Non-responders
ORR: Objective response rate
PCoA: Principal coordinate analysis
PD-1: Programmed cell death protein 1
PD-L1: Programmed cell death protein 1 ligand 1
R: Responders
RECIST 1.1: Response Evaluation Criteria in Solid Tumors 1.1
