# Supplementary figures and images for: Gut microbiome affects the response to anti-PD-1 immunotherapy in patients with hepatocellular carcinoma
Source: J Immunother Cancer. 2019 Jul 23;7:193. doi: 10.1186/s40425-019-0650-9 (PMC6651993; doi:10.1186/s40425-019-0650-9)

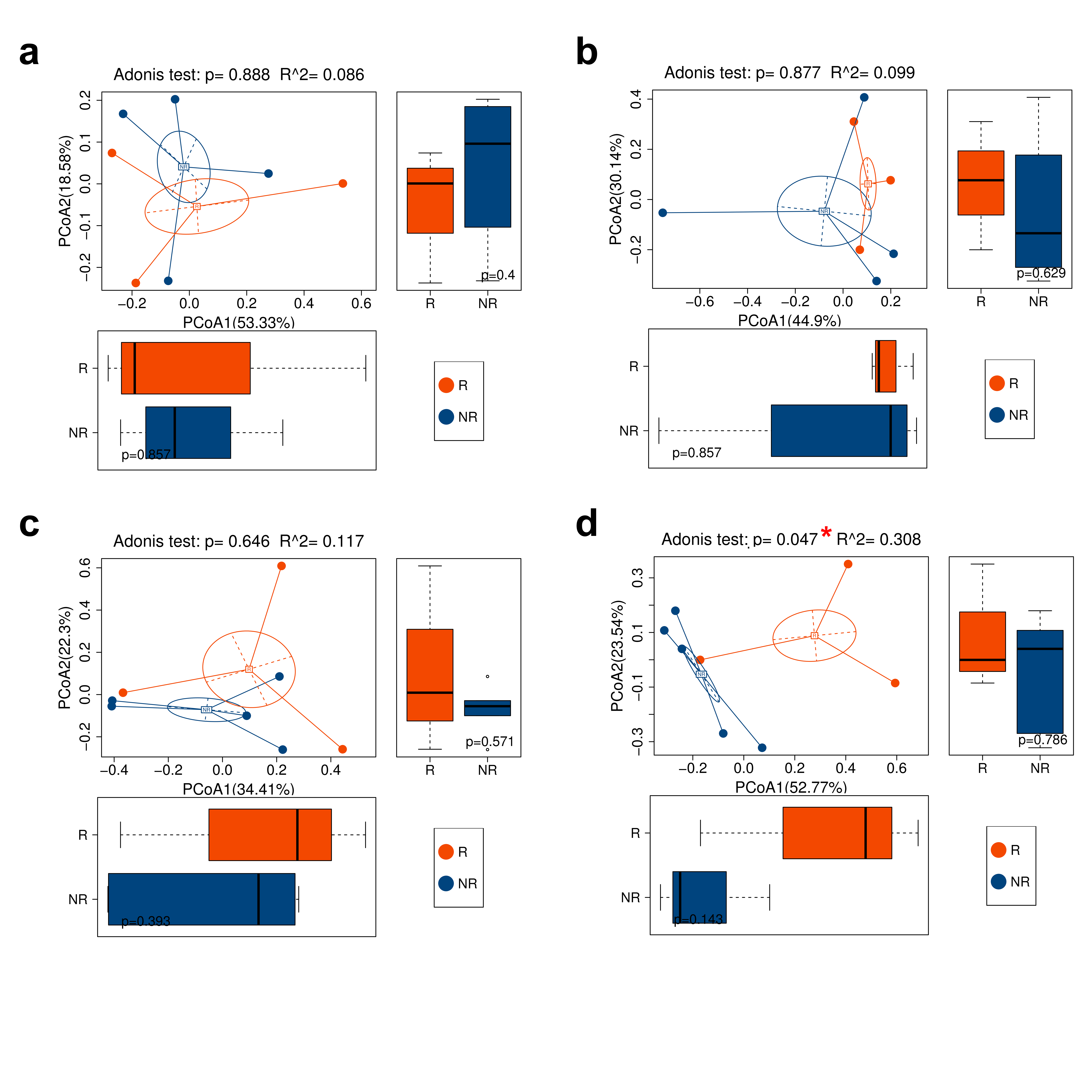

Supplement: Supplementary file 1 — Figure S1. Principal componeLnt analysis (PCoA) based on Bray-Curtis distances, followed by Adonis test at Day 0 (a), Week 1 (b), Week 3 (c), and Week 6 (d). (PNG 1455 kb) [file 40425_2019_650_MOESM1_ESM.png]

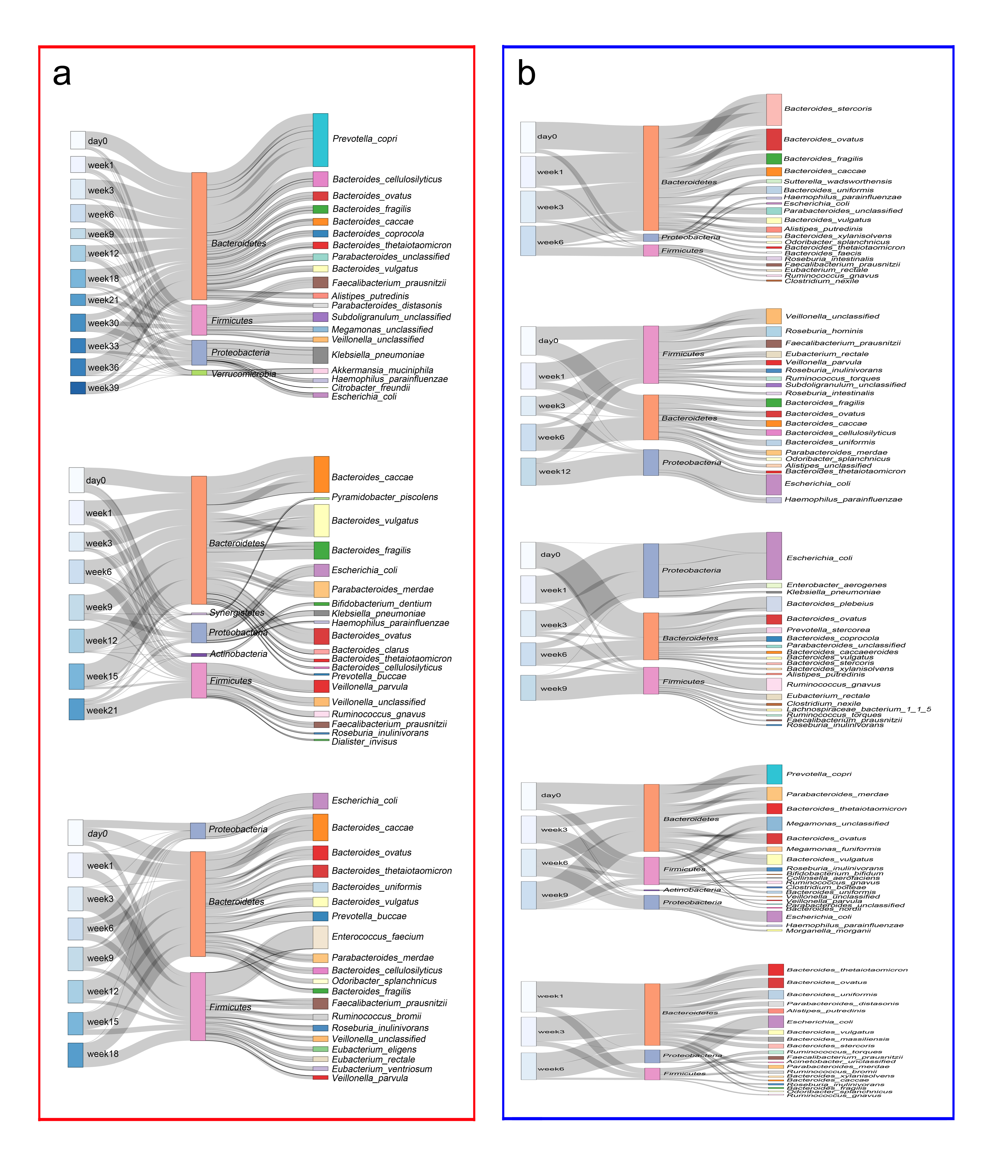

Supplement: Supplementary file 3 — Figure S3. Sankey analysis of all R and NR during the treatment. (PNG 9946 kb) [file 40425_2019_650_MOESM3_ESM.png]

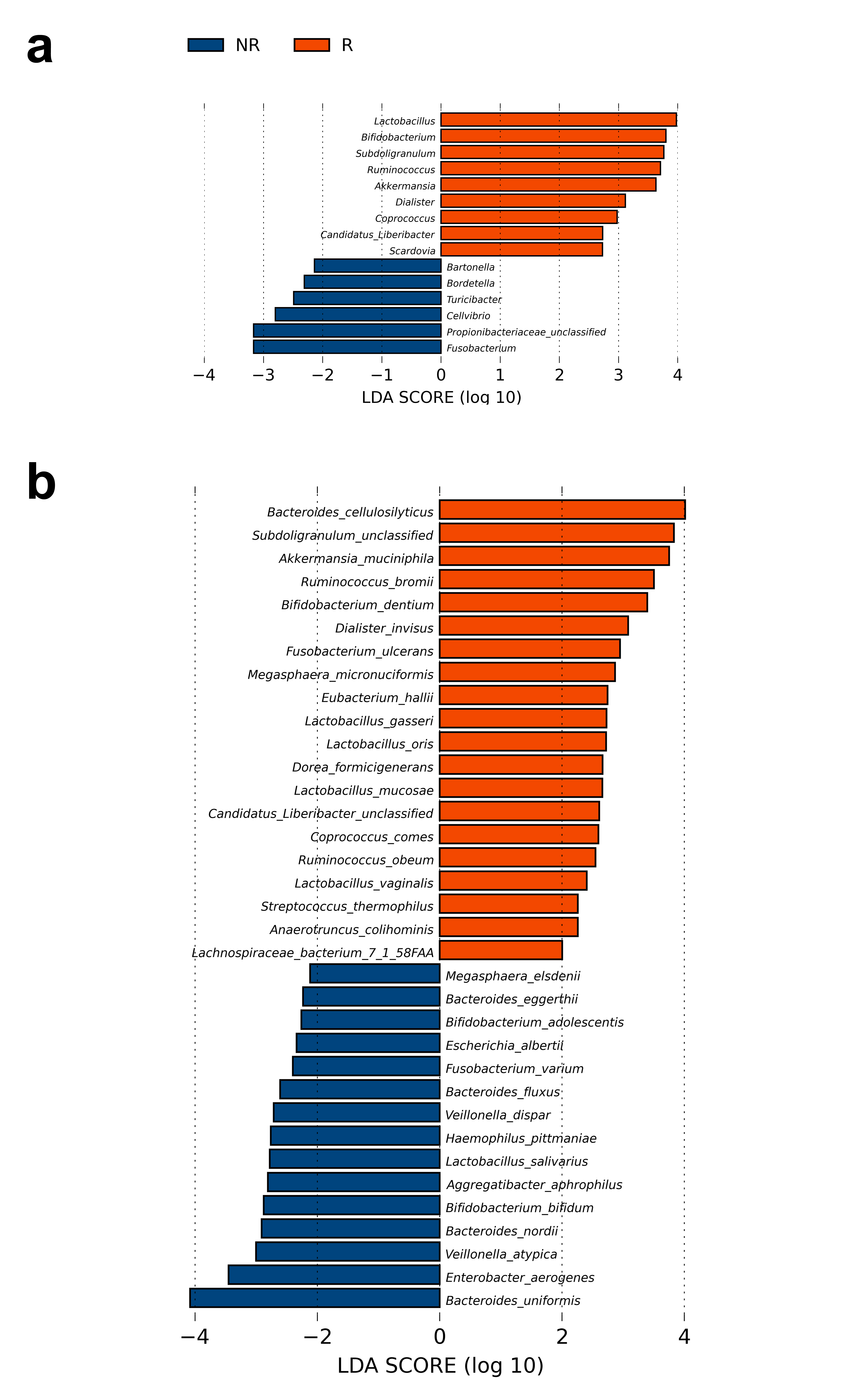

Supplement: Supplementary file 4 — Figure S4. Differentially abundant genera (a) and species (b) between R and NR, identified by LEfSe. (PNG 2863 kb) [file 40425_2019_650_MOESM4_ESM.png]

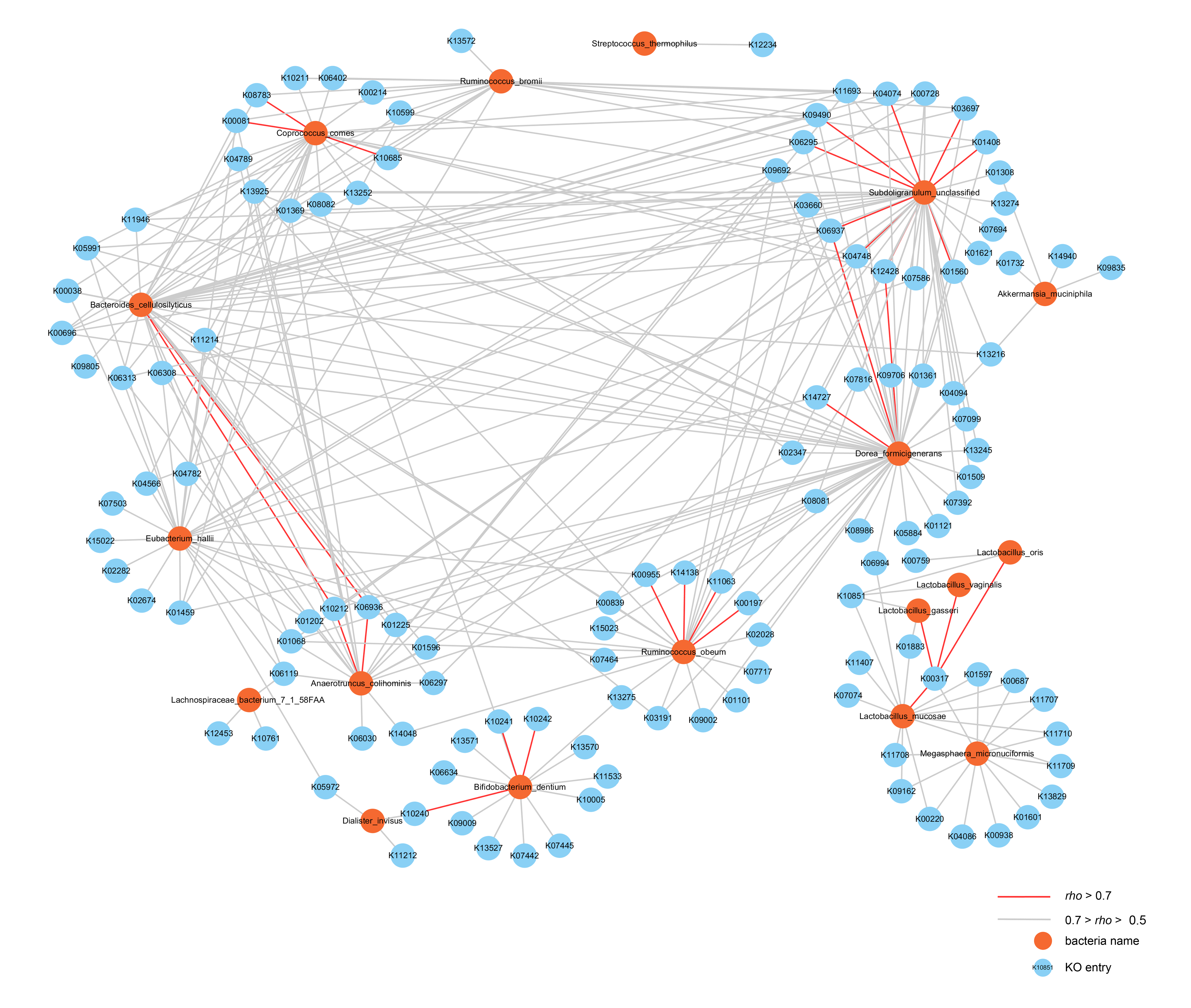

Supplement: Supplementary file 5 — Figure S5. Positive correlation network of significant R-enriched species and KOs. Correlations with rho > 0.7 are shown in red, while those with rho > 0.5 are shown in gray. (PNG 3679 kb) [file 40425_2019_650_MOESM5_ESM.png]
